# Feasibility of [^68^Ga]Ga-FAPI PET Molecular Imaging in Atherosclerosis Compared with [^18^F]FDG in Oncological Patients

**DOI:** 10.3390/diagnostics15243099

**Published:** 2025-12-05

**Authors:** Raffaella Calabretta, Ebru Atli, Barbara Katharina Geist, Dina Muin, Lucia Zisser, Clemens P. Spielvogel, Christina Falkenbach, Elisabeth Kretschmer-Chott, Stefan Schmitl, Jutta Bergler-Klein, Xiang Li, Patrick Binder, Marcus Hacker

**Affiliations:** 1 Division of Nuclear Medicine, Department of Biomedical Imaging and Image-Guided Therapy, Medical University of Vienna, 1090 Vienna, Austria; 2 Division of Cardiology, Department of Internal Medicine II, Medical University of Vienna, 1090 Vienna, Austria; jutta.bergler-klein@meduniwien.ac.at

**Keywords:** atherosclerosis, PET molecular imaging, [^68^Ga]Ga-FAPI, [^18^F]FDG, cardio-oncology

## Abstract

**Background:** Cardiovascular disease (CVD), driven primarily by atherosclerosis, is a major cause of morbidity and mortality among cancer patients. This study aims to evaluate the diagnostic value of [^68^Ga]Ga-FAPI-PET as a novel molecular imaging tool for atherosclerosis, compared with established, non-specific [^18^F]FDG. **Methods:** We retrospectively analyzed twenty patients with bladder cancer who underwent [^68^Ga]Ga-FAPI positron-emission tomography/magnetic resonance (PET/MR) and [^18^F]FDG positron emission tomography/computed tomography (PET/CT) at staging. The target-to-background ratio (TBRs) of both tracers were assessed along six arterial segments, and uptake patterns were compared between the two radiotracers. Additionally, associations between the intensity of PET-active lesions and certain CVD risk factors, as well as the intake of acetylsalicylic acid (ASA), were evaluated. **Results:** [^68^Ga]Ga-FAPI detects significantly more active arterial PET lesions and shows significantly higher uptake than [^18^F]FDG in the per-lesion analysis (TBR_FAPI_: 1.7 ± 0.5 vs. TBR_FDG_: 1.4 ± 0.2; difference 19%; *p* < 0.001) and in the patient-based analysis (TBR_FAPI_: 1.7 ± 0.4 vs. TBR_FDG_: 1.4 ± 0.2; difference 19%; *p* = 0.018). Arterial hypertension (*p* < 0.001), dyslipidemia (*p* < 0.001), and particularly type 2 diabetes mellitus (*p* < 0.001; difference 34%), were significantly associated with elevated [^68^Ga]Ga-FAPI expression compared to [^18^F]FDG uptake. ASA therapy was associated with a significant reduction in arterial [^68^Ga]Ga-FAPI expression than [^18^F]FDG (*p* = 0.02). **Conclusions:** [^68^Ga]Ga-FAPI-PET imaging, demonstrating superior detection of atherosclerotic activity compared to [^18^F]FDG, might be a promising molecular imaging marker for atherosclerosis.

## 1. Introduction

Cardiovascular disease (CVD) is highly prevalent and the leading cause of death among oncological patients. Compared with non-oncological subjects, adult cancer survivors have a growing risk of atherosclerotic CVD not related to traditional risk factors. Cancer itself, as well as its complications and anti-cancer treatments, can significantly impact CV health in cancer survivors [1]. CV imaging in cardio-oncology is thus an important tool for baseline risk assessment and detection of CVD in cancer patients receiving cardio-toxic cancer therapies [2]. A key driver of CVD is atherosclerosis, a progressive, chronic metabolic disease characterized by the accumulation of lipids, inflammatory cells, and connective tissue within the arterial wall, leading to the formation of atherosclerotic plaques. Atherosclerosis is responsible for most heart attacks, strokes, and peripheral vascular disease [3,4]. As previously described for immune checkpoint inhibitor (ICI) treatment, oncological patients with documented atherosclerosis in imaging at the time of staging present a higher risk of CV exacerbation, significantly impacting their clinical management in the field of cardio-oncology [5].

Detection of arterial inflammation with imaging is an attractive approach to identify patients at highest risk of plaque rupture, and 2-deoxy-2-[^18^F]fluoro-D-glucose ([^18^F]FDG) positron-emission tomography in combination with computed tomography (PET/CT) or magnetic resonance (PET/MR) is the standard validated and acknowledged diagnostic tool for assessing and quantifying atherosclerotic inflammation in vivo [6]. [^18^F]FDG is a glucose analogue that is taken up by cells with high glucose consumption, and its accumulation in the arterial wall is thought to reflect the presence of activated immune cells in atherosclerotic plaques [6]. Previous studies have demonstrated a correlation between [^18^F]FDG uptake and in vivo macrophage markers, but the distribution profile of [^18^F]FDG across macrophages and other arterial cells, including medial smooth muscle cells, has not been fully clarified [7,8]. Furthermore, increased [^18^F]FDG-avidity mirrors increased glucose metabolism, a typical process in patients suffering from type 2 diabetes mellitus (T2DM) [6,9]. Therefore, the biological basis of cellular [^18^F]FDG uptake is influenced by many metabolic factors and, consequently, the interpretation of the imaging findings can be confounded by these conditions.

A key component of the outermost adventitia layer of the arteries is represented by the fibroblasts. They exist in the form of numerous subtypes, even in a quiescent state. In the pathogenesis of atherosclerosis, their phenotypic and functional changes may occur before or together with endothelial activation. However, little is known about the direct involvement of activated fibroblasts in atherosclerosis development and progression [10]. The [^68^Ga]-labeled fibroblast activation protein (FAP) inhibitor ([^68^Ga]Ga-FAPI) PET tracer is based on the molecular targeting of the FAP, which is known to be highly expressed in various biological processes, including fibrosis, tumors, and wound healing. Its clinical spectrum has recently been expanded from oncology to CV medicine as well [11]. Recent research has demonstrated that [^68^Ga]Ga-FAPI PET/CT might have potential for imaging fibroblastic activation in the arterial wall, by showing that increased expression of FAP in fibrous caps may contribute to progression of atherosclerotic plaques [12]. Furthermore, another study has explained that [^68^Ga]Ga-FAPI PET imaging may identify arterial wall lesions and may correlate significantly with the calcified plaque burden but is not consistently associated with the analyzed CV risk factors. Apparently, arterial [^68^Ga]Ga-FAPI expression may be partially explained by image noise [13].

In this study, although aware of the different limitations, we aimed to quantify the arterial fibroblast activation for investigating the diagnostic value of [^68^Ga]Ga-FAPI, as a novel PET molecular non-invasive marker of atherosclerosis, compared with [^18^F]FDG, which currently represents a validated tracer in this field, in correlation with several CVD risk factors, in oncological patients.

## 2. Materials and Methods

### 2.1. Study Population

In this study, twenty patients (*n* = 20) with different histologically proven bladder cancers (15 men, 5 women; mean age 68 ± 11 years) who underwent [^68^Ga]Ga-FAPI PET/MR and [^18^F]FDG PET/CT imaging for staging 1–6 weeks before undergoing radical cystectomy, as part of a clinical prospective study (Ethics Approval No. 1238/2022), have been retrospectively analyzed. Clinical patient characteristics are summarized in Table 1.

The present research was performed in line with the principles of the Declaration of Helsinki. Approval was granted by the Ethics Committee of the Medical University of Vienna (Ethics Approval No. 2440/2024).

### 2.2. Imaging Protocols

According to the imaging protocol of the trial, all patients underwent PET/MR approximately 60 min after intravenous injection of 158 ± 23 MBq [^68^Ga]Ga-FAPI and of 20 mg furosemide on Biograph mMR^®^(Siemens. Healthcare, Germany). The pelvic MR imaging protocol, acquired over approximately 20 min, included T2 SPACE, T2 turbo spin echo (TSE), and diffusion-weighted sequences. After intravenous administration of a gadolinium-based contrast agent at a dose of 0.2 mL/kg, additional pelvic imaging was performed using an axial T1 volumetric interpolated breath-hold examination (VIBE) sequence. Whole-body imaging—from the skull base to the proximal femur—was then acquired over approximately 4 min, including T1 VIBE with fat suppression, T2 HASTE, and axial diffusion-weighted sequences with apparent diffusion coefficient (ADC) mapping (b-values: 50, 800, and 1400).

Whole-body PET images were performed from the skull base to the proximal femur approximately 60 min after intravenous injection of 205 ± 48 MBq [^18^F]FDG and of 20 mg furosemide by using a Biograph Vision^TM^ PET/CT^®^—(Siemens Healthineers, Knoxville, TN, USA). Contrast-enhanced CT was conducted after intravenous injection of approximately 100 mL iodinated contrast agent for diagnostic purposes and for attenuation correction in individuals with tolerance to the contrast agent and with normal level (>30 mg/dL) of estimated glomerular rate (eGFR). Otherwise, a non-contrast-enhanced CT was performed. Additionally, late image acquisition of the pelvis was performed.

### 2.3. Imaging Analysis

An experienced nuclear medicine physician using state-of-the-art imaging software by Hermes Medical Solutions (Stockholm, Sweden; released version 2.18) evaluated the images. A whole-segment analysis was performed on both scans, and subsequently, the corresponding segments were compared. In more detail, [^68^Ga]Ga-FAPI and [^18^F]FDG maximum standardized uptake values (SUV_max_) were derived by placing 1 cm^3^ volume of interest (VOI) along six artery segments (ascending, descending, abdominal aorta, aortic arch, and iliac arteries). For bloodpool correction, 1 cm^3^ VOI was placed within the lumen of the superior vena cava, and the mean SUV was calculated as SUV_bloodpool_. Target-to-background ratios (TBRs) were derived by correcting SUV_max_ values for SUV_bloodpool_, as previously described, to make them comparable between patients and scans [6,14]. According to the current literature, a TBR threshold > 1.6 was considered significant for identifying the arterial active segments [6].

Since we would like to investigate the diagnostic value of [^68^Ga]Ga-FAPI, we identified 374 arterial lesions with active inflammation on [^68^Ga]Ga-FAPI PET and uptake patterns were compared between both radiotracers. Additionally, we evaluated the association of CVD risk factors, such as arterial hypertension, T2DM, and dyslipidemia (under statin treatment *N* = 8), and of CV-specific therapy by acetylsalicylic acid (ASA) intake (100 mg/day) with arterial TBRs.

### 2.4. Statistical Analysis

Each lesion was annotated with dichotomous parameters (yes/no) available from clinical data, particularly hypertension, T2DM, and ASA intake. Lesion TBRs were separated according to the presented groups, and mean ± standard deviation (SD) and differences between groups (in percent) were calculated, whereby statistical significances were evaluated with Student’s *t*-test and, in the case of comparisons between tracers, with the paired Student’s *t*-test. A two-sided *p*-value of <0.05 was considered statistically significant. For subgroup comparisons, a Bonferroni correction was applied, and thus, *p* < 0.01 was considered significant.

## 3. Results

**Figure 1:**
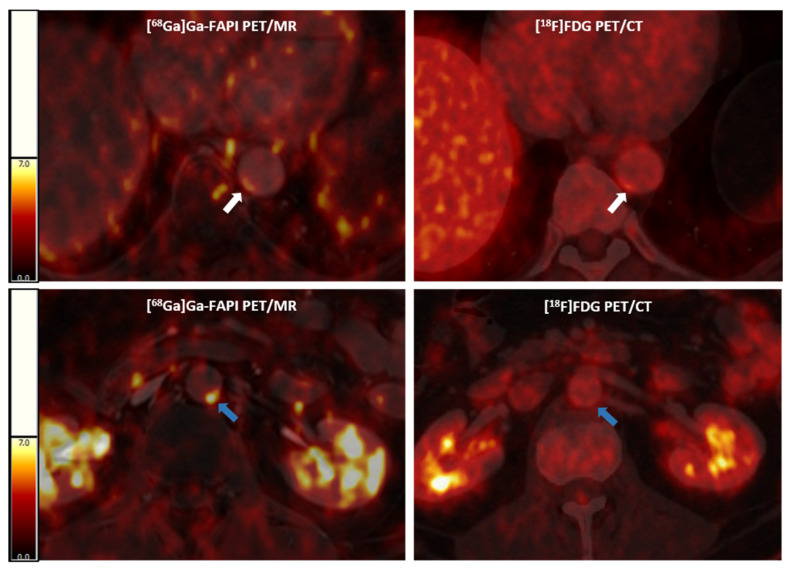
Representative [^68^Ga]Ga-FAPI PET/MR (left panels) and [^18^F]FDG PET/CT (right panels) images. Above, an arterial lesion in the thoracic aorta (white arrows) with higher [^68^Ga]Ga-FAPI expression and lower [^18^F]FDG uptake; below, an arterial lesion (blue arrows) with a higher [^68^Ga]Ga-FAPI expression and without significant [^18^F]FDG activity in a cancer patient. [^68^Ga]Ga-FAPI, [^68^Ga]-labeled fibroblast activation protein inhibitor; [^18^F]FDG, 2-deoxy-2-[^18^F]fluoro-D-glucose; PET/CT, positron emission tomography/computer tomography; PET/MR, positron emission tomography/magnetic resonance.

[^68^Ga]Ga-FAPI consistently identifies more active vascular lesions and exhibits significantly higher arterial uptake than [^18^F]FDG, as shown representatively in Figure 1.

**Figure 2:**
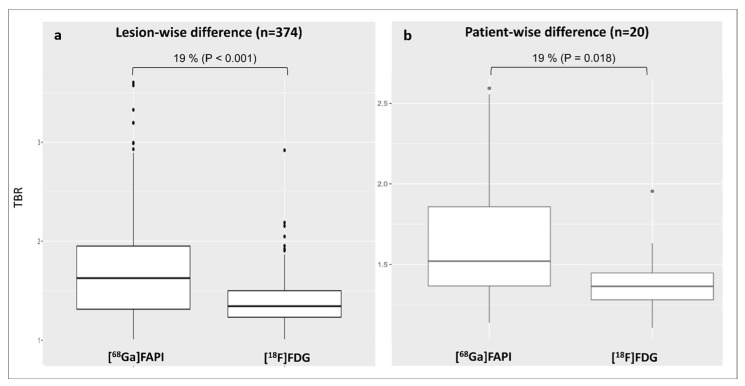
Distribution of arterial lesional TBR values for both tracers according to the per-lesion analysis (**a**) and patient-based analysis (**b**). [^68^Ga]Ga-FAPI, [^68^Ga]-labeled fibroblast activation protein inhibitor; [^18^F]FDG, 2-deoxy-2-[^18^F]fluoro-D-glucose; TBR, target-to-background ratio.

In the per-lesions analysis, [^68^Ga]Ga-FAPI identified more active lesions and showed a 19% higher uptake than [^18^F]FDG (TBR_FAPI_: 1.7 ± 0.5 vs. TBR_FDG_: 1.4 ± 0.2; *p* < 0.001) (Figure 2a). In the patient-based analysis, TBR_FAPI_ was significantly higher (+19%) compared to TBR_FDG_ (TBR_FAPI_: 1.7 ± 0.4 vs. TBR_FDG_: 1.4 ± 0.2, *p* = 0.02) (Figure 2b).

Interestingly, across the different arterial segments, [^68^Ga]Ga-FAPI identified more active lesions and showed significantly higher uptake than [^18^F]FDG in all segments except the iliac arteries (Table 2).

**Figure 3:**
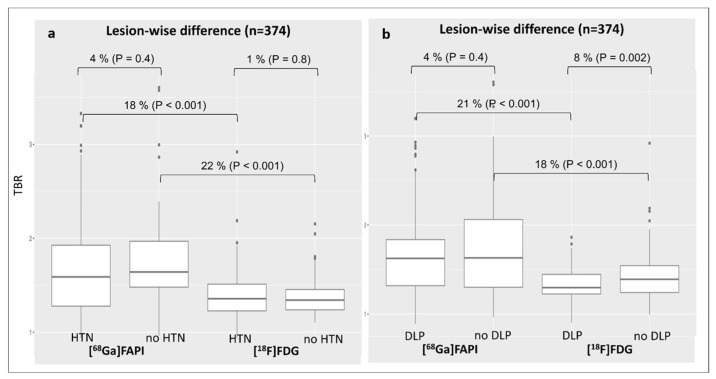
Changes in lesional TBR values for both tracers according to the diagnosis of arterial hypertension (**a**) or dyslipidemia (**b**). DLP, dyslipidemia; [^68^Ga]Ga-FAPI, [^68^Ga]-labeled fibroblast activation protein inhibitor; [^18^F]FDG, 2-deoxy-2-[^18^F]fluoro-D-glucose; HNT, arterial hypertension; TBR, target-to-background ratio.

The presence of arterial hypertension (TBR_FAPI_: 1.7 ± 0.6 vs. 1.4 ± 0.3, *p* < 0.001—Figure 3a) or dyslipidemia (TBR_FAPI_: 1.7 ± 0.5 vs. 1.3 ± 0.2, *p* < 0.001—Figure 3b) was significantly associated with [^68^Ga]Ga-FAPI overexpression compared to [^18^F]FDG activity. In contrast, no significant differences in [^68^Ga]Ga-FAPI expression were found when comparing subjects suffering from arterial hypertension (TBR_FAPI_: 1.7 ± 0.6 vs. 1.8 ± 0.5, *p* = 0.4—Figure 3a) or dyslipidemia (TBR_FAPI_: 1.7 ± 0.5 vs. 1.8 ± 0.6, *p* = 0.3—Figure 3b) to those without, respectively.

**Figure 4:**
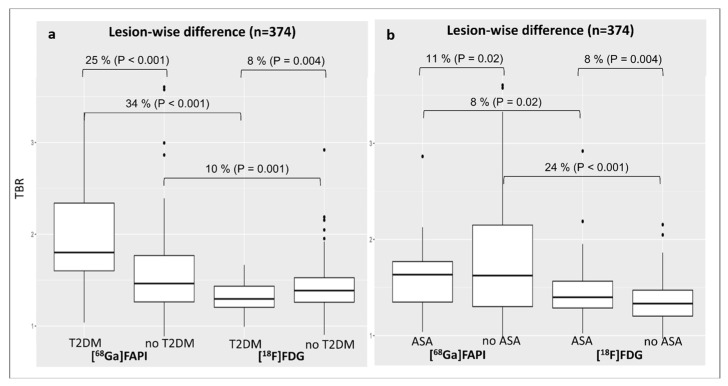
Changes in lesional TBR values for both tracers according to the diagnosis of T2DM (**a**) or the intake of ASA (**b**). ASA, acetylsalicylic acid; [^68^Ga]Ga-FAPI, [^68^Ga]-labeled fibroblast activation protein inhibitor; [^18^F]FDG, 2-deoxy-2-[^18^F]fluoro-D-glucose; TBR, target-to-background ratio; T2DM, type 2 diabetes mellitus.

The superiority of TBR_FAPI_ over TBR_FDG_ was especially pronounced in patients with T2DM (TBR_FAPI_: 2.0 ± 0.6 vs. TBR_FDG_: 1.3 ± 0.2; difference 34%, *p* < 0.001) (Figure 4a). Remarkably, in contrast to arterial [^18^F]FDG uptake (TBR_FDG_: 1.3 ± 0.2 vs. 1.4 ± 0.3, difference 8%; *p* < 0.001), [^68^Ga]Ga-FAPI expression was 25% higher in diabetic patients as compared to non-diabetic patients (TBR_FAPI_: 2.0 ± 0.6 vs. 1.6 ± 0.5, *p* < 0.001) (Figure 4a).

Concerning ASA therapy, patients not treated with ASA showed a 24% higher arterial [^68^Ga]Ga-FAPI expression than [^18^F]FDG (TBR_FAPI_: 1.8 ± 0.6 vs. TBR_FDG_: 1.3 ± 0.2, *p* < 0.001), whereas the difference between TBR_FAPI_ and TBR_FDG_ was lower, although not significant in patients under ASA treatment (TBR_FAPI_: 1.6 ± 0.3 vs. 1.5 ± 0.3; difference 8%, *p* = 0.02) (Figure 4b). Interestingly, subjects under ASA therapy presented a lower [^68^Ga]Ga-FAPI expression compared with patients without ASA treatment (TBR_FAPI_: 1.6 ± 0.3 vs. 1.8 ± 0.6; difference 1%, *p* = 0.02) (Figure 4b).

In a multivariable linear regression including hypertension and dyslipidemia, both T2DM and ASA intake (Table 3) remained independently associated with arterial [^68^Ga]Ga-FAPI uptake (both *p* < 0.001), confirming their strong and independent contribution beyond traditional CV risk factors. For [^18^F]FDG, dyslipidemia and ASA intake remained significant independent predictors of arterial TBR (both *p* < 0.001), whereas the association with T2DM was attenuated but persisted at borderline significance (*p* = 0.04) after multivariable adjustment.

To account for clustering of lesions within patients, we fitted mixed-effects models with a random intercept for each patient. For [^68^Ga]Ga-FAPI, between-patient variance was substantial (0.174), and the association with T2DM remained significant in the mixed-effects framework, consistent with the patient-level multivariable analysis. For [^18^F]FDG, between-patient variance was minimal (0.034), and none of the risk factors reached significance in the mixed model. Nevertheless, patient-level analyses showed that the trends for T2DM and ASA persisted (*p* = 0.25), albeit weaklier. Given the lower sensitivity of FDG for arterial inflammation, these secondary findings do not alter the main tracer comparison.

## 4. Discussion

To the best of our knowledge, this is the first study exploring the potential diagnostic value of [^68^Ga]Ga-FAPI PET molecular imaging in atherosclerosis in comparison to the established standard tracer for vascular activity [^18^F]FDG, among cancer patients. We systematically compared arterial uptake of [^68^Ga]Ga-FAPI with [^18^F]FDG PET imaging and demonstrated that [^68^Ga]Ga-FAPI consistently identifies a higher number of active arterial lesions and exhibits significantly higher uptake than [^18^F]FDG in both per-lesion and patient-based analyses. The 19% increase in arterial TBR observed with [^68^Ga]Ga-FAPI suggests that fibroblast activation, a key component of vascular remodeling and inflammation, might have a higher sensitivity for the presence of atherosclerosis compared to glucose consumption, as it is measured with [^18^F]FDG.

In patients with T2DM, [^68^Ga]Ga-FAPI expression was especially high (34%) when compared to [^18^F]FDG, as reflected by both absolute and relative terms. Additionally, diabetic patients demonstrated 25% higher [^68^Ga]Ga-FAPI expression compared to non-diabetic subjects. This underscores the relevance of fibroblast activation in diabetic vasculopathy and reinforces the tracer’s potential utility in detecting subclinical vascular inflammation in this high-risk population compared to [^18^F]FDG. [^18^F]FDG is a glucose analogue taken up by cells with high metabolic activity, including activated immune cells within atherosclerotic plaques, reflecting vascular inflammation and plaque vulnerability [6,15]. Currently, [^18^F]FDG-PET is a validated diagnostic and functional imaging tool for assessing and quantifying atherosclerotic inflammation [6,16]. However, the biological basis of cellular [^18^F]FDG uptake is influenced by many metabolic factors, and it is known that this tracer presents several limitations in atherosclerotic PET imaging. Previous studies have demonstrated a correlation between [^18^F]FDG uptake and in vivo macrophage markers, but the distribution profile of [^18^F]FDG across macrophages and other arterial cells, including medial smooth muscle cells, has not been fully clarified, resulting in decreased specificity of imaging [7,17,18]. Overall, whilst remaining a valid diagnostic tool, careful consideration of T2DM’s impact on [^18^F]FDG imaging results is crucial for accurate diagnosis and management [6,19].

On the other hand, [^68^Ga]Ga-FAPI, targeting activated fibroblasts involved in plaque remodeling and fibrosis, might be a possible new valid alternative in the field of PET imaging in atherosclerosis, particularly in diabetic individuals, potentially by capturing distinct biological processes. A particularly intriguing aspect of our results concerns the modulation of [^68^Ga]Ga-FAPI expression by acetylsalicylic acid therapy. While arterial TBR remained significantly higher with [^68^Ga]Ga-FAPI versus [^18^F]FDG in both treated and untreated patients, the magnitude of the difference was attenuated in the ASA-treated group.

Moreover, [^68^Ga]Ga-FAPI expression alone was significantly lower in patients on ASA therapy, potentially mirroring the anti-inflammatory effect of this drug, particularly on activated fibroblasts.

### Study Limitations

This study, however, presents several limitations. First, the relatively small sample size limits the statistical power and generalizability of the results. Second, the retrospective design introduces potential selection bias and limits control over confounding variables. Prospective studies with standardized imaging time points and protocols, possibly using the same imaging techniques, would strengthen the evidence. Other limitations are related to physical and technical issues. Importantly, as previously described, the use of different hybrid PET techniques, such as PET/MR for [^68^Ga]Ga-FAPI and PET/CT for [^18^F]FDG, might introduce inherent differences in SUV measurements between the two imaging modalities and, therefore, adversely affect the validity of the results. However, a significant correlation has been demonstrated between the two techniques in the identification of pathological lesions [20]. Furthermore, prior research suggested considering the possibility that elevated arterial wall uptake based on [^68^Ga]Ga-FAPI SUV_max_ might be partially explained by image noise [13]. Another technical issue to consider is the potential partial-volume effect of PET images, resulting from limited spatial resolution and differences in reconstruction methods between PET/CT and PET/MI imaging. Several established methods are routinely used to correct the partial-volume effect in PET images, improving both the accuracy of radioactivity measurements and the quality of the final image. Finally, the absence of histopathological validation limits the ability to definitively link imaging findings with underlying biological processes, emphasizing the need for future studies with tissue correlation.

## 5. Conclusions

The findings of this study are consistent with the emerging literature supporting the use of [^68^Ga]Ga-FAPI beyond oncology, including cardiac fibrosis and inflammatory CVD [11,21]. Our preliminary data supports the feasibility of [^68^Ga]Ga-FAPI PET molecular imaging in atherosclerosis as a complement to the established tracer [^18^F]FDG, suggesting its potential clinical relevance for assessing the modulation of vascular inflammation, particularly in routinely performed oncological investigations and in metabolically compromised populations, such as patients with T2DM. The different effects of comorbidities and co-medication on lesional [^68^Ga]Ga-FAPI and [^18^F]FDG uptake might provide unique insights into plaque biology, potentially improving CV risk assessment and stratification in cancer patients, who are at elevated risk for CVD due to both their disease and its treatments.

## 6. Future Directions

Considering our results and the study limitations, a prospective trial including a larger and more diverse patient cohort undergoing [^68^Ga]Ga-FAPI PET/MR or PET/CT is necessary to validate these preliminary observations and to better characterize the diagnostic accuracy of [^68^Ga]Ga-FAPI in atherosclerosis. Additional correlation with clinical CV outcomes, as well as with histopathology, would enhance our understanding of fibroblast activation in atherosclerotic plaques and may offer potential advantages over the non-specific [^18^F]FDG.

## Figures and Tables

**Table 1:** Clinical patient characteristics.

Clinical Characteristics	*N*	%
Gender (males/females)	15/5	75/25
Age (years)	68 ± 11	-
BMI (kg/m^2^)	26 ± 4	-
Hypertension	12	60
Dyslipidemia	1	50
T2DM	7	35
Smoking	9	45
CAD	2	10
Family history CAD	0	0
Prior myocardial infarction	1	5
Prior TIA/Stroke	2	10
Heart failure	0	0
Atrial fibrillation	1	5
PAD	1	5
COPD	3	15
Antidiabetic treatments	6	30
Metformin	2	10
SGLT-2-Inhibitor + Metformin	1	5
DPP-4-Inhibitors	1	5
DPP-4-Inhibitors + Metformin	1	5
Insulin	1	5
Acetylsalicylic acid	7	35
Statins	8	40
Beta-blockers	6	30
ACE inhibitors/ARBs	4	20
CCBs	0	0
Diagnosis of MIBC	12	60
Diagnosis of NMIBC	2	10
Diagnosis of UCB	6	30
Diagnosis of MI-NEC	2	10

Abbreviations. ACE: angiotensin-converting enzyme; ARBs: angiotensin receptor blockers; BMI: body mass index; CAD: coronary artery disease; CCBs; calcium channel blockers; DPP-4: Dipeptidyl peptidase-4; MIBC: muscle-invasive bladder cancer; MI-NEC: muscle-infiltrating neuroendocrine carcinoma; NMIBC: non-muscle-invasive bladder cancer; PAD: peripheral artery disease; SGLT-2: sodium glucose transporter-2; TIA: transient ischemic attack; T2DM: Type 2 diabetes mellitus; COPD: chronic obstructive pulmonary disease; UCB: urothelial carcinoma of the bladder.

**Table 2:** Distribution of lesional TBR values for both tracers in all arterial segments.

	[^68^Ga]Ga-FAPI	[^18^F]FDG	Difference [^68^Ga]Ga-FAPI Versus [^18^F]FDG
Aortic Arch	1.7 ± 0.5	1.4 ± 0.2	19% (*p* = 0.001 *)
Aorta Ascendens	1.6 ± 0.4	1.4 ± 0.2	14% (*p* = 0.001 *)
Aorta Descendens	1.8 ± 0.6	1.4 ± 0.3	21% (*p* < 0.001 *)
Abdominal Aorta	1.8 ± 0.6	1.4 ± 0.3	21% (*p* = 0.001 *)
Iliac Arteries	1.6 ± 0.7	1.4 ± 0.3	13% (*p* = 0.4)

Uptake of [^68^Ga]Ga-FAPI and [^18^F]FDG measured in all investigated arterial segments is presented as mean value plus/minus one standard deviation. Differences between [^68^Ga]Ga-FAPI and [^18^F]FDG are listed in the last column and shown as percentages, along with the corresponding *p*-values from Student’s *t*-test. * Significant values. Abbreviations. [^68^Ga]Ga-FAPI, [^68^Ga]-labeled fibroblast activation protein inhibitor; [^18^F]FDG, 2-deoxy-2-[^18^F]fluoro-D-glucose; TBR, target-to-background ratio.

**Table 3:** Multivariable linear regression including the selected CV risk factors.

		[^68^Ga]Ga-FAPI	[^18^F]FDG	Cohen’s d [^68^Ga]Ga-FAPI Versus [^18^F]FDG
Arterial hypertension	Yes	1.7 ± 0.6	1.4 ± 0.3	0.4 (0.2–0.6) *
	No	1.8 ± 0.5	1.4 ± 0.2	0.9 (0.6–1.1) *
	Cohen’s d	0.1 (−0.2–0.4)	−0.0 (−0.3–0.2)	
Dyslipidemia	Yes	1.7 ± 0.5	1.3 ± 0.2	0.6 (0.4–0.9) *
	No	1.8 ± 0.6	1.4 ± 0.3	0.5 (0.3–0.7) *
	Cohen’s d	0.1 (−0.1–−0.4) *	0.5 (0.2–0.7) *	
T2DM	Yes	2.0 ± 0.6	1.3 ± 0.2	0.3 (0.1–0.5) *
	No	1.6 ± 0.5	1.4 ± 0.3	1.0 (0.7–1.3) *
	Cohen’s d	−0.8 (−1.1–−0.5) *	0.4 (0.1–0.7) *	
ASA	Yes	1.6 ± 0.3	1.5 ± 0.3	0.7 (0.5–0.9) *
	No	1.8 ± 0.6	1.3 ± 0.2	0.3 (0.0–0.5) *
	Cohen’s d	0.4 (0.1–−0.7)	−0.5 (−0.8–−0.2) *	

Uptake of [^68^Ga]Ga-FAPI and [^18^F]FDG in patients, divided into various subgroups according to the presence/absence of CV risk factors, is presented as mean value plus/minus one standard deviation. Differences are reported using Cohen’s d (95% confidence interval). Differences between [^68^Ga]Ga-FAPI and [^18^F]FDG within the same subgroup are listed in the last column. * Significant values. Abbreviations. ASA, acetylsalicylic acid; CV, cardiovascular; [^68^Ga]Ga-FAPI, [^68^Ga]-labeled fibroblast activation protein inhibitor; [^18^F]FDG, 2-deoxy-2-[^18^F]fluoro-D-glucose; T2DM, type 2 diabetes mellitus.

## Data Availability

The original contributions presented in this study are included in this article. Further inquiries can be directed to the corresponding author.

## Abbreviations

The following abbreviations are used in this manuscript:

ADC	Apparent diffusion coefficient
ASA	Acetylsalicylic acid
CV	Cardiovascular
CVD	Cardiovascular disease
CT	Computer tomography
dL	Deciliter
eGFR	Estimated glomerular filtration rate
[^68^Ga]Ga-FAPI	[^68^Ga]-labeled fibroblast activation protein inhibitor
[^18^F]FDG	2-deoxy-2-[^18^F]fluoro-D-glucose
ICI	Immune checkpoint inhibitor
kg	Kilogram
MBq	Mega Becquerel
mg	Milligram
mL	Milliliter
MR	Magnetic resonance
PET	Positron-emission tomography
SD	Standard deviation
SUV	Standardized uptake value
TBR	Target-to-background ratio
TSE	Turbo spin echo
T2DM	Type 2 diabetes mellitus
VIBE	Volumetric interpolated breath-hold examination
VOI	Volume of interest
